# Combined effects of electric toothbrushing and dentifrice on artificial stain removal: An *in vitro* study

**DOI:** 10.4317/jced.54312

**Published:** 2018-03-01

**Authors:** Akiko Haruyama, Atsushi Kameyama, Tomoko Ono, Yukako Baba, Toshiko Sugiyama, Setsuko Sugiyama, Toshiyuki Takahashi

**Affiliations:** 1 Senior Assistant Professor, Department of Operative Dentistry, Cariology and Pulp Biology, Tokyo Dental College, 2-9-18 Misaki-cho, Chiyoda-ku, Tokyo, Japan; 2 Associate Professor, Department of Operative Dentistry, Cariology and Pulp Biology, Tokyo Dental College, 1-2-2 Masago, Mihama-ku, Chiba, Japan; 3 Former Student, Tokyo Dental College School of Dental Hygiene, 1-2-2 Masago, Mihama-ku, Chiba, Japan; 4 Senior Assistant Professor, Division of General Dentistry, Tokyo Dental College Chiba Hospital, 1-2-2 Masago, Mihama-ku, Chiba, Japan; 5 Assistant Professor, Division of General Dentistry, Tokyo Dental College Chiba Hospital, 1-2-2 Masago, Mihama-ku, Chiba, Japan; 6 Associate Professor and Head, Division of General Dentistry, Tokyo Dental College Chiba Hospital, 1-2-2 Masago, Mihama-ku, Chiba, Japan

## Abstract

**Background:**

This *in vitro* study aimed to clarify the combined effect of electric toothbrushing and dentifrice on the removal of artificial stain.

**Material and Methods:**

Twenty-five bovine incisors were cut at the cervix and the crown was embedded in auto-cured acrylic resin. Specimens were abraded using #240 SiC paper to obtain a flat enamel surface, and 20 specimens were treated with 10% citric acid / 3% ferric chloride solution followed by 1% tannic acid solution to produce surface staining. They were divided into four groups: 1) brushing with an electric toothbrush and whitening dentifrice (group S+B); 2) brushing with an electric toothbrush and fluoride dentifrice (group S+C); 3) brushing with an electric toothbrush and no dentifrice (group S); and 4) no brushing (control group). The remaining five specimens were used as a baseline. Color values (L*, a*, and b* were measured before brushing (0 min), and at 1 min, 5 min, 10 min, and 20 min using a microscopic area spectrophotometer. The color change (*ΔE*) was calculated by subtracting the baseline values from the final color values obtained at each time point. The data were statistically analyzed using two-way repeated-measures analysis of variance and Tukey’s honest significant difference test as a post hoc test (*p*<0.05).

**Results:**

The L* values of groups S+B and S+C increased over time (*p*<0.05), but no significant differences were observed in group S and the control group at any of the time points (*p*>0.05). Groups S+B and S+C demonstrated greater ΔE values than group S.

**Conclusions:**

The combination of electric toothbrushing and dentifrice removed the artificial stain more effectively than brushing without dentifrice. However, the stain removal was limited. The two dentifrices evaluated in this study exhibited similar stain removal effects.

** Key words:**Color change, stain removal, dentifrice, electric toothbrush, whitening effect.

## Introduction

Extrinsic stain is linked with the adsorption of colored agents into the pellicle on the surface of enamel, and is caused by poor brushing technique, tobacco smoking, tea drinking, dietary intake of colored foods (such as red wine or spicy foods), the use of cationic antiseptics such as chlorhexidine and cetylpyridinium chloride (CPC), or ingestion of metal salts such as iron and tin (1,2). Adsorption of stain is one factor in the deterioration of dental esthetics.

Professional dental prophylaxis is known to remove extrinsic stain efficaciously (3). Although professional prophylaxis by a dental hygienist removes staining, the procedure incurs a cost each time. Self-brushing at home is also a simple and effective method of stain removal with lower costs than professional prophylaxis. Self-brushing with an electric toothbrush has been reported to be superior to manual toothbrushing for stain removal (4).

Dentifrices contain abrasives that assist in removing plaque and stains from the tooth surface (5). Contemporary over-the-counter dentifrices incorporate additional effects for improving or maintaining oral health: caries/erosion prevention by fluoride, efficient decomposition of plaque by enzymes, control of gingival inflammation, prevention of calculus formation, reduction of dentin hypersensitivity, and prevention of oral malodor. In recent years, whitening dentifrices, which contain additives to improve esthetics, have also become available (1).

The cleaning action of a dentifrice is mainly promoted by abrasive particles, such as softened hydrated silica, calcium carbonate and dicalcium phosphate dihydrate, which are designed to dislodge dental plaque and stains (6). Contemporary whitening dentifrices generally contain higher amounts of abrasive particles than conventional dentifrices (1,7), as well as active chemical agents (1). Whitening dentifrices containing sodium pyrophosphate are available in Japan, but their stain removal capability has not yet been determined (8).

Therefore, the purpose of this *in vitro* study was to clarify the combined effect of electric toothbrushing and two contemporary over-the-counter dentifrices on the removal of artificial stains. The null hypotheses tested was that the stain removal effect of the combined use of electric toothbrushing and over-the-counter dentifrices is the same as brushing with an electric toothbrush alone.

## Material and Methods

-Preparation of specimens

Twenty extracted bovine teeth, frozen to maintain freshness, were defrosted and cut at the cervix using a diamond cut saw (KT100, Maruto Instrument, Tokyo, Japan). The dental pulp tissue was removed from the crown portions using a #80 K-file (Mani, Tokyo, Japan). The pulp cavity was then filled with auto-cured acrylic resin (Unifast III, GC, Tokyo), and the crown was embedded in an acrylic ring (Refine Tech, Yokohama, Japan) using epoxy resin (Scandiprex, Fritsch Japan, Yokohama, Japan). The embedded specimens were abraded with #240 SiC paper to obtain a flat enamel surface using an automatic polishing machine (Automet 250, Buehler, IL, USA).

-Initial color measurement (baseline)

The initial color of each specimen was measured with a microscopic area spectrophotometer (MSP-300H, Nippon Denshoku Industries, Tokyo, Japan), using the CIE L*a*b* color system, where the L* axis indicates the value (lightness/darkness), the a* axis represents the redness (+a*) or the greenness (-a*), and the b* axis demonstrates the yellowness (+b*) or the blueness (-b*) (L*baseline, a*baseline, b*baseline). Color was measured five times for each specimen, and the average values obtained were defined as the color values of each specimen.

-Artificial staining procedure

The prepared enamel surfaces were treated with an aqueous solution of 10 wt% citric acid / 3 wt% ferric chloride (10-3 PRE-treating agent, Nippon Shiken Corporation, Tokyo, Japan) for 60 s and then thoroughly rinsed with water spray. The specimens were immersed in chicken egg white for 10 min and then immersed in a 1 wt% aqueous solution of tannic acid (Wako Pure Chemicals, Osaka, Japan). Upon completion of the staining process, the specimens were rinsed with running tap water, and stored in 37 °C water for 1 week. After storage, the color of each specimen was measured again (L*0 min, a*0 min, b*0 min).

-Brushing protocol

Specimens were randomly divided into four groups (n=5).

- Group S+B: Brushing with an electric toothbrush (Sonicare Easy Clean, Phillips Oral Healthcare, Bothell, WA, USA) and Brilliant More® whitening dentifrice (Lion Dental Products, Tokyo, Japan)

- Group S+C: Brushing with an electric toothbrush (Sonicare Easy Clean) and Clinica fluoride-containing dentifrice (Lion, Tokyo, Japan)

- Group S: Brushing with an electric toothbrush (Sonicare Easy Clean) with no dentifrice.

- Control group: No brushing.

Each specimen was fixed onto a disposable dish with double-sided tape and put onto a kitchen scale. A small amount of tap water and a rice-grain-size amount of dentifrice were placed onto the central portion of the enamel surface and the specimen was brushed with hand pressure to maintain a loading of 90 gf. The brushing procedure continued for a total of 20 minutes, with color measurements taken at 1 min, 4 min, 5 min, 10 min, and 20 min for each specimen. After the brushing procedure, the specimens were washed with water spray and dried with delicate task wipe towels (KimWipes, Nippon Paper Crecia, Tokyo, Japan).

-Color change measurements

The color of each specimen was measured at 1 min (L*1 min, a*1 min, b*1 min), 5 min (L*5 min, a*5 min, b*5 min), 10 min (L*10 min, a*10 min, b*10 min), and after the completion of brushing (L*20 min, a*20 min, b*20 min). Color was measured five times for each specimen, and the average values obtained were defined as the color values of each specimen. Next, the differences between the L*, a*, and b* values at each time point and the values before brushing were calculated as follows:

ΔL* = L*1/5/10/20 min- L*0 min

Δa* = a*1/5/10/20 min- a*0 min

Δb* = b*1/5/10/20 min- b*0 min

**Figure 1:**
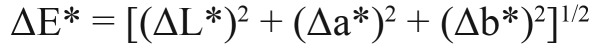
Equation.

The color change (ΔE*) was calculated using the following equation, (Fig. 1).

**Figure 2:**
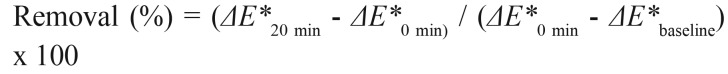
Formula.

The percentage of stain removal was calculated as follows, (Fig. 2).

-Statistical analysis

The obtained data were used to calculate the mean and standard deviation (SD) for each group, and were statistically analyzed using two-way repeated measures analysis of variance (ANOVA), followed by Tukey’s HSD test with statistical significance set at a *p*-value of 0.05. All statistical analyses were performed using IBM SPSS statistics 18 for Windows (IBM Japan Inc., Tokyo, Japan).

## Results

The results of the changes in individual color parameters are shown in  Table 1, and the color change (ΔE*) results are shown in  Table 2. The two-way ANOVA in the L* value revealed a significant interaction between pair of means for factors “group” (*p*=0.000) and “brushing time” (*p*=0.000). There was a significant interaction between the independent variables of these two factors (*p*=0.000). Groups S+B and S+C both showed a significant increase in the L* value at 5 min and later (*p*<0.05). However, no significant changes were found in the L* value of group S and the control group (*p*>0.05). No significant differences were found between groups S+B and S+C, or between group S and the control group (*p*>0.05).

**Table 1:**
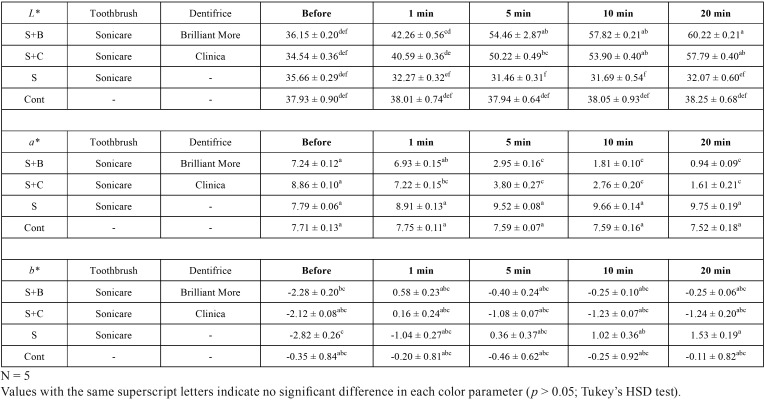
Means and standard deviations for L* values, a* values and b* values.

**Table 2:**
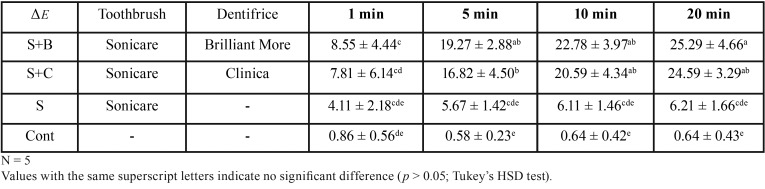
Means and standard deviations for *ΔE* values.

The two-way ANOVA in the a* value revealed a significant interaction between pair of means for factors “group” (*p*=0.000) and “brushing time” (*p*=0.000). There was a significant interaction between the independent variables of these two factors (*p*=0.000). Groups S+B and S+C both showed a significant reduction in a* value at 5 min and later (*p*<0.05). However, no significant changes in a* value were found in group S and the control group (*p*>0.05). No significant differences were found between groups S+B and S+C, or between group S and the control group (*p*>0.05).

The two-way ANOVA in the b* value revealed a significant interaction between pair of means for factors “group” (*p*=0.000) and “brushing time” (*p*=0.000). There was a significant interaction between the independent variables of these two factors (*p* = 0.000). No significant differences were found in the b* value at any time point in groups S+B and S+C or the control group (*p*>0.05). However, a significant increase in b* value was detected at 10 min and 20 min in group S (p<0.05).

Groups S+B and S+C both exhibited gradual changes in ΔE* value and this difference was significant at 5 min (p<0.05). However, no significant differences were detected among the 5 min, 10 min and 20 min time points in any of the four groups (p>0.05).

The percentage removal of stains is shown in  Table 3. Groups S+B and S+C both exhibited higher stain removal scores than group S. There was no significant difference between the stain removal scores of S+B and S+C (*p*>0.05).

**Table 3:**
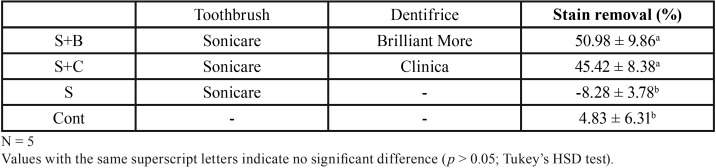
Stain removal efficacy (mean ± SD; %).

## Discussion

The purpose of this *in vitro* study was to clarify the combined effect of electric toothbrushing and two contemporary over-the-counter dentifrices on the removal of artificial stains. We demonstrated that the combined use of the dentifrices and an electric toothbrush (Sonicare) removes stains more effectively than brushing with the Sonicare toothbrush alone. Therefore, the null hypothesis of this study was rejected.

Aqueous solutions of tea or instant coffee have commonly been used to stain the tooth substrate (8). This method is appropriate for *in vitro* comparison of the effects of bleaching, because bleaching agents affect not only the applied surface staining but also the discolored dentinal substrate. Additionally, these staining methods have been reported to gradually increase the darkness, making it difficult to stabilize the color level (5). However, to evaluate the effect of toothbrushing on extrinsic stain removal, it is necessary to create an artificial stain on the enamel surface only, and to avoid penetration of the pigment into the tooth as much as possible.

Ferric ion (III) combines with tannic acid and forms brown or black ferric tannate on contact with air (9). This study applied this phenomenon to form artificial extrinsic stain on the enamel surface. We applied an aqueous solution of 10% citric acid / 3% ferric chloride (10-3 solution), which is commonly used for preconditioning of 4-META/MMA-TBB resin, onto the enamel surface, and then immersed it in an aqueous solution of tannic acid. This method effectively formed an artificial stain on the enamel surface, in which the L* value changed from 85.32 ± 0.33 to 36.07 ± 1.31, the a* value changed from -2.14 ± 0.03 to 7.90 ± 0.63, and the b* value changed from 3.95 ± 0.26 to -1.89 ± 0.99. We determined in the pilot study that the artificial stain formed by this method was confined to the enamel surface layer, because the stain disappeared easily following brief acid etching of the surface. Additionally, no changes in L*, a*, b* were observed during the evaluating period. Therefore, our method is effective for examining the effect of extrinsic stain removal from the tooth surface *in vitro*.

This study involved brushing the stained enamel surface for 1, 5, 10 and 20 min using a Sonicare Easy Clean electric toothbrush. The Sonicare toothbrush instructions suggest brushing the whole dentition for 2 min each time (10,11), which equates to brushing each tooth surface for approximately 2 s. Therefore, brushing the specimens for 1, 5, 10 and 20 min corresponds to normal brushing for approximately 10 days, 50 days, 3 months and 6 months, respectively.

The Sonicare toothbrush has been reported to remove plaque more effectively than a manual toothbrush (12,13). However, this study found no significant change in the color of the specimens when no dentifrice was used when compared with the control. Dawson *et al.* (9) reported that brushing with a manual toothbrush removed almost no extrinsic stain from the surface of a hydroxyapatite disc stained with ferric tannate. Other reports found that the stain removal effect of Sonicare was no different to that of a conventional manual brush (14). Although it may be effective in removing plaque, mechanical brushing without any dentifrice might be less effective in removing extrinsic stain that has penetrated the pellicle and is strongly bound to it.

The brightness of the teeth significantly increased with the combination of Sonicare toothbrushing and either of the dentifrices (Brilliant More or Clinica). In addition, the combination of Sonicare toothbrushing and dentifrice contributed to a reduction in redness. Both dentifrices contain anhydrous silica as an abrasive. During tooth brushing, abrasive particles become trapped between the toothbrush and the stained tooth surface, thereby efficiently removing extrinsic stain (4,15).

Phosphate materials, such as sodium pyrophosphate, sodium tripolyphosphate and sodium hexametaphosphate have a strong binding affinity for calcium ions, and this adsorption effect desorbs stain components (16,17). Addition of these ingredients can also be expected to assist in the prevention of calculus formation (18). It has been reported that dentifrices containing sodium pyrophosphate have a significantly higher stain removal effect than non-whitening silica-contained control dentifrices (19). In this study, the whitening dentifrice containing sodium pyrophosphate tended to be more effective in removing extrinsic stains than sodium-pyrophosphate-free dentifrice, but no statistically significant difference was found between them. Our results therefore support the current opinion that the primary stain removal ingredient in dentifrice is the abrasive (1), and that sodium pyrophosphate supplements the effect of the abrasive.

Several *in vivo* and *in vitro* whitening studies have reported that a yellow to blue color shift (i.e. reduction in b*) is important in aiding overall self-perception of tooth whiteness (19-21). However, this study did not find a reduction in b* when dentifrice was used. Blending of blue covarine into the silica-based dentifrice has been reported to contribute to a reduction in b* value (22,23). Another study reported that the reduction in b* value in teeth brushed with a silica-based dentifrice was significantly lower than in teeth that underwent in-office bleaching using 35% hydrogen peroxide and at-home bleaching using 10% carbamide peroxide (24). These findings support the results of our study.

Within the limitations of this *in vitro* study, it can be concluded that the combination of dentifrice and electric toothbrushing removed artificial staining more effectively than electric brushing without dentifrice. However, we found no significant difference between the effectiveness of whitening dentifrice containing sodium pyrophosphate and fluoride dentifrice in the removal of artificial extrinsic stain.
